# Tetra-μ-chlorido-bis­(18-crown-6)platinum(II)dipotassium(I)

**DOI:** 10.1107/S1600536810016648

**Published:** 2010-05-19

**Authors:** B. Ravindran Durai Nayagam, Samuel Robinson Jebas, D. Kalavathy, R. Murugesan, Dieter Schollmeyer

**Affiliations:** a Department of Chemistry, Popes College, Sawyerpuram 628 251, India; b Department of Physics, Sethupathy Government Arts College, Ramanathapuram 623 502, Tamilnadu, India; c Department of Physics, Popes College, Sawyerpuram 628 251, India; d Department of Chemistry, T.D.M.N.S. College, T. Kallikulam, Tamilnadu, India; e Institut für Organische Chemie, Universität Mainz, Duesbergweg 10-14, 55099 Mainz, Germany

## Abstract

In the title compound, [K_2_PtCl_4_(C_12_H_24_O_6_)_2_], the Pt^II^ ion is located on an inversion centre and is coordinated by four Cl atoms, forming a square-planar geometry. The K^I^ ion is coordinated by six O atoms of the crown ether and two bridging Cl atoms. The K^I^ ion is displaced by 0.756 (2) Å from the mean plane of the six O atoms of the crown ether. The mol­ecules are connected by weak C—H⋯O hydrogen bonds, forming an infinite two-dimensional network parallel to the (10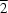) plane. Intra- and inter­molecular C—H⋯Cl hydrogen bonds are also observed.

## Related literature

For bond-length data, see: Allen *et al.* (1987 ▶). For the biological activity of metal platinum derivatives, see: Loehrer *et al.* (1988 ▶); Weiss & Christian (1993 ▶).
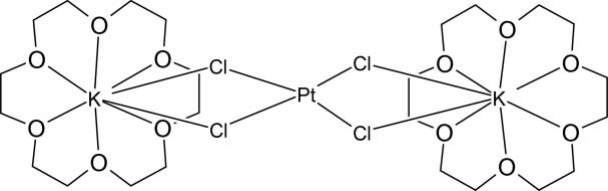


## Experimental

### 

#### Crystal data

[K_2_PtCl_4_(C_12_H_24_O_6_)_2_]
*M* *_r_* = 943.71
Monoclinic, 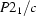

*a* = 11.6307 (6) Å
*b* = 8.4715 (4) Å
*c* = 19.1586 (9) Å
β = 107.3248 (11)°
*V* = 1802.05 (15) Å^3^
*Z* = 2
Mo *K*α radiation
μ = 4.47 mm^−1^
*T* = 173 K
0.39 × 0.37 × 0.08 mm

#### Data collection

Bruker SMART APEXII CCD diffractometer
Absorption correction: multi-scan (*SADABS*; Bruker, 2008 ▶) *T*_min_ = 0.409, *T*_max_ = 0.699
15436 measured reflections
4252 independent reflections
3659 reflections with *I* > 2σ(*I*)
*R*_int_ = 0.036

#### Refinement

*R*[*F* ^2^ > 2σ(*F* ^2^)] = 0.020
*wR*(*F* ^2^) = 0.053
*S* = 1.04
4252 reflections
196 parameters
H-atom parameters constrained
Δρ_max_ = 1.36 e Å^−3^
Δρ_min_ = −0.84 e Å^−3^

### 

Data collection: *APEX2* (Bruker, 2008 ▶); cell refinement: *APEX2*; data reduction: *APEX2*; program(s) used to solve structure: *SHELXS97* (Sheldrick, 2008 ▶); program(s) used to refine structure: *SHELXL97* (Sheldrick, 2008 ▶); molecular graphics: *SHELXTL* (Sheldrick, 2008 ▶); software used to prepare material for publication: *SHELXTL* and *PLATON* (Spek, 2009 ▶).

## Supplementary Material

Crystal structure: contains datablocks global, I. DOI: 10.1107/S1600536810016648/is2544sup1.cif

Structure factors: contains datablocks I. DOI: 10.1107/S1600536810016648/is2544Isup2.hkl

Additional supplementary materials:  crystallographic information; 3D view; checkCIF report

## Figures and Tables

**Table 1:** Hydrogen-bond geometry (Å, °)

*D*—H⋯*A*	*D*—H	H⋯*A*	*D*⋯*A*	*D*—H⋯*A*
C5—H5*B*⋯O10^i^	0.99	2.57	3.318 (3)	133
C11—H11*A*⋯Cl11^ii^	0.99	2.80	3.678 (3)	148
C17—H17*A*⋯Cl11	0.99	2.81	3.643 (3)	142

Symmetry codes: (i) 
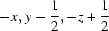
; (ii) 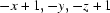.

**Fig. 1.**
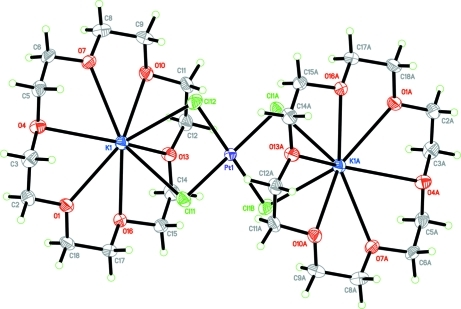
The molecular structure of the title compound, showing 50% probability displacement ellipsoids and the atom numbering scheme. The suffix A corresponds to symmetry code (-x + 1, -y, -z + 1).

**Fig. 2.**
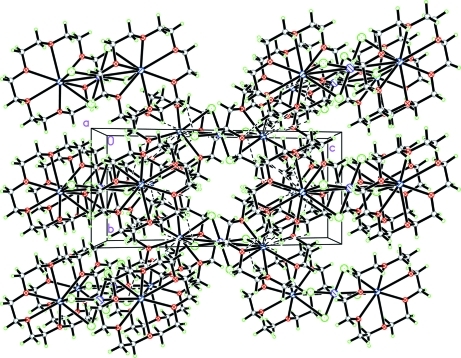
The crystal packing of the title compound, showing an infinite two dimensional network along the plane (102).

## supplementary crystallographic information

### Comment

The platinum complex, *cis*-diamminedichloroplatinum(II) (cisplatin), is one of the most widely used antitumour drugs in the world (Weiss & Christian, 1993; Loehrer *et al.*, 1988). Due to the importance of the metal platinum, we report here the crystal structure of the title compound, (I).

The bond lengths and bond angles in (Fig. 1) are within normal ranges (Allen *et al.*, 1987). The Pt^II^ ion exhibits a distorted square planar coordination geometry. The geometry is completed by four chorine atoms. The K^+^ ion is coordinated by six oxygen atoms of the crown ether and also by two terminal Cl atom attached to the metal platinum, forming a eight-fold coordination. The O atoms are oriented towards the centre of the crown ether cavity and O—C—C—O fragments have alternate +SC and –SC conformations. The K^+^ cation is displaced by 0.756 (2) Å from the mean plane of the six oxygen atoms of the crown ether.

The crystal packing is consolidated by C—H···Cl and weak C—H···O hydrogen bonds. The molecules form an infinite two dimensional network parallel to the (102) plane through the C—H···O hydrogen bonds.

### Experimental

A mixture of potassium tetrachloroplatinate(II) (K_2_PtCl_4_; 0. 0208 g, 0.05 mmol) and 18-crown-6 ether (0.026 g, 0.1 mmol) in acetone and benzene (5/5 ml) was heated at 313 K with stirring for 30 min. The yellow colour solution was allowed to undergo slow evaporation. Fine orange crystals are formed after three days.

### Refinement

All H atoms were positioned geometrically (C—H = 0.99 Å) and refined using a riding model, with *U*_iso_(H) = 1.2*U*_eq_(C). The highest peak in the difference Fourier map is located 1.07 Å from atom Pt1.

### Crystal data

**Table:**

[K_2_PtCl_4_(C_12_H_24_O_6_)_2_]	*F*(000) = 944
*M**_r_* = 943.71	*D*_x_ = 1.739 Mg m^−^^3^
Monoclinic, *P*2_1_/*c*	Mo *K*α radiation, λ = 0.71073 Å
Hall symbol: -P 2ybc	Cell parameters from 8198 reflections
*a* = 11.6307 (6) Å	θ = 2.6–27.8°
*b* = 8.4715 (4) Å	µ = 4.47 mm^−^^1^
*c* = 19.1586 (9) Å	*T* = 173 K
β = 107.3248 (11)°	Plate, orange
*V* = 1802.05 (15) Å^3^	0.39 × 0.37 × 0.08 mm
*Z* = 2	

### Data collection

**Table:**

Bruker SMART APEXII CCD diffractometer	4252 independent reflections
Radiation source: sealed Tube	3659 reflections with *I* > 2σ(*I*)
graphite	*R*_int_ = 0.036
ω scan	θ_max_ = 27.8°, θ_min_ = 1.8°
Absorption correction: multi-scan (*SADABS*; Bruker, 2008)	*h* = −11→15
*T*_min_ = 0.409, *T*_max_ = 0.699	*k* = −11→10
15436 measured reflections	*l* = −24→25

### Refinement

**Table:**

Refinement on *F*^2^	Primary atom site location: structure-invariant direct methods
Least-squares matrix: full	Secondary atom site location: difference Fourier map
*R*[*F*^2^ > 2σ(*F*^2^)] = 0.020	Hydrogen site location: inferred from neighbouring sites
*wR*(*F*^2^) = 0.053	H-atom parameters constrained
*S* = 1.04	*w* = 1/[σ^2^(*F*_o_^2^) + (0.0229*P*)^2^ + 0.3938*P*] where *P* = (*F*_o_^2^ + 2*F*_c_^2^)/3
4252 reflections	(Δ/σ)_max_ < 0.001
196 parameters	Δρ_max_ = 1.36 e Å^−^^3^
0 restraints	Δρ_min_ = −0.84 e Å^−^^3^

### Special details

**Table:**

Geometry. All esds (except the esd in the dihedral angle between two l.s. planes) are estimated using the full covariance matrix. The cell esds are taken into account individually in the estimation of esds in distances, angles and torsion angles; correlations between esds in cell parameters are only used when they are defined by crystal symmetry. An approximate (isotropic) treatment of cell esds is used for estimating esds involving l.s. planes.
Refinement. Refinement of *F*^2^ against ALL reflections. The weighted *R*-factor wR and goodness of fit *S* are based on *F*^2^, conventional *R*-factors *R* are based on *F*, with *F* set to zero for negative *F*^2^. The threshold expression of *F*^2^ > σ(*F*^2^) is used only for calculating *R*-factors(gt) etc. and is not relevant to the choice of reflections for refinement. *R*-factors based on *F*^2^ are statistically about twice as large as those based on *F*, and *R*- factors based on ALL data will be even larger.

### Fractional atomic coordinates and isotropic or equivalent isotropic displacement parameters (Å^2^)

**Table:**

	*x*	*y*	*z*	*U*_iso_*/*U*_eq_	
Pt1	0.5000	0.0000	0.5000	0.01602 (5)	
Cl11	0.54452 (6)	0.02672 (7)	0.39113 (3)	0.02592 (13)	
Cl12	0.41425 (5)	−0.24295 (6)	0.46094 (3)	0.02923 (14)	
K1	0.26519 (5)	−0.03222 (6)	0.32365 (3)	0.02257 (11)	
O1	0.30996 (16)	0.0581 (2)	0.19194 (9)	0.0281 (4)	
C2	0.2692 (3)	−0.0325 (3)	0.12656 (15)	0.0303 (6)	
H2A	0.3292	−0.0280	0.0992	0.036*	
H2B	0.1922	0.0108	0.0950	0.036*	
C3	0.2521 (2)	−0.1987 (3)	0.14642 (15)	0.0309 (6)	
H3A	0.2285	−0.2648	0.1018	0.037*	
H3B	0.3285	−0.2405	0.1794	0.037*	
O4	0.16047 (14)	−0.20461 (18)	0.18204 (9)	0.0248 (4)	
C5	0.1404 (2)	−0.3633 (3)	0.20022 (14)	0.0283 (5)	
H5A	0.2162	−0.4091	0.2321	0.034*	
H5B	0.1135	−0.4278	0.1552	0.034*	
C6	0.0464 (2)	−0.3648 (3)	0.23892 (14)	0.0284 (5)	
H6A	−0.0265	−0.3084	0.2095	0.034*	
H6B	0.0237	−0.4749	0.2461	0.034*	
O7	0.09465 (14)	−0.28863 (19)	0.30812 (9)	0.0246 (4)	
C8	0.0148 (2)	−0.2960 (3)	0.35139 (14)	0.0288 (5)	
H8A	−0.0051	−0.4073	0.3586	0.035*	
H8B	−0.0609	−0.2394	0.3267	0.035*	
C9	0.0756 (2)	−0.2208 (3)	0.42348 (14)	0.0282 (5)	
H9A	0.0269	−0.2367	0.4574	0.034*	
H9B	0.1557	−0.2693	0.4454	0.034*	
O10	0.08862 (15)	−0.0570 (2)	0.41216 (9)	0.0254 (4)	
C11	0.1541 (2)	0.0232 (3)	0.47782 (14)	0.0262 (5)	
H11A	0.2357	−0.0229	0.4971	0.031*	
H11B	0.1120	0.0111	0.5154	0.031*	
C12	0.1632 (2)	0.1943 (3)	0.46078 (13)	0.0273 (5)	
H12A	0.0816	0.2389	0.4392	0.033*	
H12B	0.2029	0.2530	0.5063	0.033*	
O13	0.23094 (14)	0.21061 (18)	0.41091 (9)	0.0236 (3)	
C14	0.2572 (2)	0.3712 (3)	0.39951 (14)	0.0298 (6)	
H14A	0.2893	0.4253	0.4472	0.036*	
H14B	0.1826	0.4261	0.3714	0.036*	
C15	0.3480 (2)	0.3772 (3)	0.35834 (13)	0.0272 (5)	
H15A	0.3687	0.4883	0.3515	0.033*	
H15B	0.4225	0.3219	0.3863	0.033*	
O16	0.29863 (14)	0.30278 (19)	0.28855 (8)	0.0236 (3)	
C17	0.3846 (2)	0.3012 (3)	0.24896 (13)	0.0266 (5)	
H17A	0.4588	0.2467	0.2780	0.032*	
H17B	0.4061	0.4108	0.2399	0.032*	
C18	0.3329 (2)	0.2174 (3)	0.17747 (13)	0.0273 (5)	
H18A	0.2572	0.2693	0.1490	0.033*	
H18B	0.3905	0.2215	0.1485	0.033*	

### Atomic displacement parameters (Å^2^)

**Table:**

	*U*^11^	*U*^22^	*U*^33^	*U*^12^	*U*^13^	*U*^23^
Pt1	0.01708 (7)	0.01474 (7)	0.01610 (7)	0.00102 (4)	0.00471 (5)	−0.00062 (4)
Cl11	0.0269 (3)	0.0326 (3)	0.0206 (3)	0.0012 (2)	0.0107 (2)	0.0011 (2)
Cl12	0.0358 (3)	0.0177 (3)	0.0297 (3)	−0.0051 (2)	0.0027 (3)	−0.0029 (2)
K1	0.0248 (2)	0.0219 (2)	0.0211 (3)	−0.00083 (19)	0.0071 (2)	−0.00075 (19)
O1	0.0399 (10)	0.0250 (8)	0.0213 (9)	−0.0081 (8)	0.0118 (8)	−0.0018 (7)
C2	0.0367 (14)	0.0370 (14)	0.0213 (13)	−0.0061 (11)	0.0147 (11)	−0.0064 (11)
C3	0.0316 (13)	0.0306 (13)	0.0328 (14)	0.0011 (10)	0.0132 (11)	−0.0107 (11)
O4	0.0261 (8)	0.0193 (8)	0.0309 (9)	0.0000 (6)	0.0112 (7)	−0.0024 (7)
C5	0.0355 (13)	0.0200 (11)	0.0265 (13)	−0.0018 (10)	0.0044 (11)	−0.0054 (10)
C6	0.0291 (13)	0.0220 (12)	0.0290 (13)	−0.0086 (9)	0.0010 (10)	−0.0006 (10)
O7	0.0228 (8)	0.0271 (8)	0.0241 (9)	−0.0059 (7)	0.0075 (7)	−0.0039 (7)
C8	0.0262 (12)	0.0230 (12)	0.0408 (15)	−0.0018 (9)	0.0155 (11)	0.0009 (11)
C9	0.0323 (13)	0.0245 (12)	0.0325 (14)	0.0018 (10)	0.0168 (11)	0.0076 (11)
O10	0.0289 (9)	0.0223 (8)	0.0227 (9)	0.0011 (7)	0.0041 (7)	0.0031 (7)
C11	0.0274 (12)	0.0347 (13)	0.0170 (12)	0.0018 (10)	0.0072 (10)	0.0020 (10)
C12	0.0320 (13)	0.0310 (13)	0.0212 (12)	0.0010 (10)	0.0113 (10)	−0.0065 (10)
O13	0.0309 (9)	0.0193 (8)	0.0234 (8)	−0.0004 (6)	0.0123 (7)	−0.0019 (7)
C14	0.0446 (15)	0.0185 (11)	0.0271 (14)	0.0013 (10)	0.0117 (12)	−0.0030 (10)
C15	0.0358 (13)	0.0193 (11)	0.0251 (13)	−0.0070 (10)	0.0068 (11)	−0.0024 (10)
O16	0.0256 (8)	0.0266 (8)	0.0187 (8)	−0.0045 (7)	0.0069 (7)	−0.0010 (7)
C17	0.0290 (12)	0.0272 (12)	0.0249 (13)	−0.0063 (10)	0.0103 (10)	0.0025 (10)
C18	0.0350 (13)	0.0274 (12)	0.0225 (12)	−0.0037 (10)	0.0130 (11)	0.0037 (10)

### Geometric parameters (Å, °)

**Table:**

Pt1—Cl11^i^	2.3050 (6)	O7—C8	1.419 (3)
Pt1—Cl11	2.3050 (6)	C8—C9	1.494 (3)
Pt1—Cl12^i^	2.3121 (5)	C8—H8A	0.9900
Pt1—Cl12	2.3121 (5)	C8—H8B	0.9900
Cl11—K1	3.1581 (8)	C9—O10	1.419 (3)
Cl12—K1	3.2242 (8)	C9—H9A	0.9900
K1—O13	2.7535 (16)	C9—H9B	0.9900
K1—O1	2.8296 (18)	O10—C11	1.433 (3)
K1—O7	2.8965 (16)	C11—C12	1.497 (3)
K1—O16	2.9687 (17)	C11—H11A	0.9900
K1—O4	3.0014 (17)	C11—H11B	0.9900
K1—O10	3.0351 (18)	C12—O13	1.414 (3)
O1—C18	1.419 (3)	C12—H12A	0.9900
O1—C2	1.425 (3)	C12—H12B	0.9900
C2—C3	1.487 (4)	O13—C14	1.425 (3)
C2—H2A	0.9900	C14—C15	1.496 (4)
C2—H2B	0.9900	C14—H14A	0.9900
C3—O4	1.427 (3)	C14—H14B	0.9900
C3—H3A	0.9900	C15—O16	1.434 (3)
C3—H3B	0.9900	C15—H15A	0.9900
O4—C5	1.426 (3)	C15—H15B	0.9900
C5—C6	1.493 (4)	O16—C17	1.424 (3)
C5—H5A	0.9900	C17—C18	1.500 (3)
C5—H5B	0.9900	C17—H17A	0.9900
C6—O7	1.431 (3)	C17—H17B	0.9900
C6—H6A	0.9900	C18—H18A	0.9900
C6—H6B	0.9900	C18—H18B	0.9900
			
Cl11^i^—Pt1—Cl11	180.0	O4—C5—H5A	109.9
Cl11^i^—Pt1—Cl12^i^	89.20 (2)	C6—C5—H5A	109.9
Cl11—Pt1—Cl12^i^	90.80 (2)	O4—C5—H5B	109.9
Cl11^i^—Pt1—Cl12	90.80 (2)	C6—C5—H5B	109.9
Cl11—Pt1—Cl12	89.20 (2)	H5A—C5—H5B	108.3
Cl12^i^—Pt1—Cl12	180.0	O7—C6—C5	108.24 (19)
Cl11^i^—Pt1—K1^i^	58.669 (18)	O7—C6—H6A	110.0
Cl11—Pt1—K1^i^	121.331 (18)	C5—C6—H6A	110.0
Cl12^i^—Pt1—K1^i^	60.300 (17)	O7—C6—H6B	110.0
Cl12—Pt1—K1^i^	119.700 (17)	C5—C6—H6B	110.0
Cl11^i^—Pt1—K1	121.331 (18)	H6A—C6—H6B	108.4
Cl11—Pt1—K1	58.669 (18)	C8—O7—C6	112.15 (17)
Cl12^i^—Pt1—K1	119.700 (17)	C8—O7—K1	121.37 (13)
Cl12—Pt1—K1	60.300 (17)	C6—O7—K1	120.09 (13)
K1^i^—Pt1—K1	180.0	O7—C8—C9	108.00 (19)
Pt1—Cl11—K1	82.76 (2)	O7—C8—H8A	110.1
Pt1—Cl12—K1	81.170 (19)	C9—C8—H8A	110.1
O13—K1—O1	115.95 (5)	O7—C8—H8B	110.1
O13—K1—O7	113.84 (5)	C9—C8—H8B	110.1
O1—K1—O7	114.43 (5)	H8A—C8—H8B	108.4
O13—K1—O16	58.38 (5)	O10—C9—C8	108.6 (2)
O1—K1—O16	57.87 (5)	O10—C9—H9A	110.0
O7—K1—O16	145.64 (5)	C8—C9—H9A	110.0
O13—K1—O4	145.00 (5)	O10—C9—H9B	110.0
O1—K1—O4	57.21 (5)	C8—C9—H9B	110.0
O7—K1—O4	57.39 (5)	H9A—C9—H9B	108.4
O16—K1—O4	107.80 (4)	C9—O10—C11	112.47 (18)
O13—K1—O10	57.87 (5)	C9—O10—K1	105.89 (13)
O1—K1—O10	148.41 (5)	C11—O10—K1	100.74 (14)
O7—K1—O10	56.55 (5)	O10—C11—C12	108.8 (2)
O16—K1—O10	110.42 (5)	O10—C11—H11A	109.9
O4—K1—O10	108.14 (5)	C12—C11—H11A	109.9
O13—K1—Cl11	87.16 (4)	O10—C11—H11B	109.9
O1—K1—Cl11	83.24 (4)	C12—C11—H11B	109.9
O7—K1—Cl11	138.60 (4)	H11A—C11—H11B	108.3
O16—K1—Cl11	75.72 (3)	O13—C12—C11	109.4 (2)
O4—K1—Cl11	122.55 (4)	O13—C12—H12A	109.8
O10—K1—Cl11	124.54 (4)	C11—C12—H12A	109.8
O13—K1—Cl12	93.34 (4)	O13—C12—H12B	109.8
O1—K1—Cl12	132.86 (4)	C11—C12—H12B	109.8
O7—K1—Cl12	81.60 (4)	H12A—C12—H12B	108.2
O16—K1—Cl12	129.73 (4)	C12—O13—C14	112.59 (18)
O4—K1—Cl12	116.29 (4)	C12—O13—K1	123.21 (13)
O10—K1—Cl12	78.16 (4)	C14—O13—K1	122.96 (13)
Cl11—K1—Cl12	61.045 (18)	O13—C14—C15	109.26 (19)
O13—K1—Pt1	65.66 (4)	O13—C14—H14A	109.8
O1—K1—Pt1	121.14 (4)	C15—C14—H14A	109.8
O7—K1—Pt1	116.72 (4)	O13—C14—H14B	109.8
O16—K1—Pt1	91.47 (3)	C15—C14—H14B	109.8
O4—K1—Pt1	149.24 (3)	H14A—C14—H14B	108.3
O10—K1—Pt1	86.13 (3)	O16—C15—C14	109.10 (19)
Cl11—K1—Pt1	38.567 (13)	O16—C15—H15A	109.9
Cl12—K1—Pt1	38.529 (12)	C14—C15—H15A	109.9
C18—O1—C2	111.94 (19)	O16—C15—H15B	109.9
C18—O1—K1	121.79 (14)	C14—C15—H15B	109.9
C2—O1—K1	122.46 (15)	H15A—C15—H15B	108.3
O1—C2—C3	108.7 (2)	C17—O16—C15	110.55 (17)
O1—C2—H2A	110.0	C17—O16—K1	105.76 (13)
C3—C2—H2A	110.0	C15—O16—K1	104.60 (12)
O1—C2—H2B	110.0	O16—C17—C18	109.66 (19)
C3—C2—H2B	110.0	O16—C17—H17A	109.7
H2A—C2—H2B	108.3	C18—C17—H17A	109.7
O4—C3—C2	109.4 (2)	O16—C17—H17B	109.7
O4—C3—H3A	109.8	C18—C17—H17B	109.7
C2—C3—H3A	109.8	H17A—C17—H17B	108.2
O4—C3—H3B	109.8	O1—C18—C17	108.61 (19)
C2—C3—H3B	109.8	O1—C18—H18A	110.0
H3A—C3—H3B	108.2	C17—C18—H18A	110.0
C5—O4—C3	110.48 (18)	O1—C18—H18B	110.0
C5—O4—K1	106.86 (13)	C17—C18—H18B	110.0
C3—O4—K1	104.80 (13)	H18A—C18—H18B	108.3
O4—C5—C6	109.07 (19)		
			
Cl12^i^—Pt1—Cl11—K1	−124.86 (2)	K1—O4—C5—C6	65.2 (2)
Cl12—Pt1—Cl11—K1	55.14 (2)	O4—C5—C6—O7	−67.1 (2)
K1^i^—Pt1—Cl11—K1	180.0	C5—C6—O7—C8	−175.16 (19)
Cl11^i^—Pt1—Cl12—K1	126.21 (2)	C5—C6—O7—K1	32.5 (2)
Cl11—Pt1—Cl12—K1	−53.79 (2)	O13—K1—O7—C8	−9.37 (17)
K1^i^—Pt1—Cl12—K1	180.0	O1—K1—O7—C8	−145.94 (15)
Pt1—Cl11—K1—O13	53.14 (4)	O16—K1—O7—C8	−77.71 (18)
Pt1—Cl11—K1—O1	169.70 (4)	O4—K1—O7—C8	−150.68 (17)
Pt1—Cl11—K1—O7	−70.62 (6)	O10—K1—O7—C8	−0.74 (15)
Pt1—Cl11—K1—O16	111.20 (4)	Cl11—K1—O7—C8	105.42 (16)
Pt1—Cl11—K1—O4	−146.57 (4)	Cl12—K1—O7—C8	80.57 (15)
Pt1—Cl11—K1—O10	6.02 (5)	Pt1—K1—O7—C8	64.24 (16)
Pt1—Cl11—K1—Cl12	−42.255 (17)	O13—K1—O7—C6	140.35 (15)
Pt1—Cl12—K1—O13	−42.60 (4)	O1—K1—O7—C6	3.78 (17)
Pt1—Cl12—K1—O1	88.11 (6)	O16—K1—O7—C6	72.01 (18)
Pt1—Cl12—K1—O7	−156.22 (4)	O4—K1—O7—C6	−0.96 (14)
Pt1—Cl12—K1—O16	8.02 (5)	O10—K1—O7—C6	148.99 (17)
Pt1—Cl12—K1—O4	156.66 (4)	Cl11—K1—O7—C6	−104.86 (15)
Pt1—Cl12—K1—O10	−98.79 (4)	Cl12—K1—O7—C6	−129.71 (15)
Pt1—Cl12—K1—Cl11	42.298 (17)	Pt1—K1—O7—C6	−146.04 (14)
Cl11^i^—Pt1—K1—O13	61.30 (4)	C6—O7—C8—C9	177.23 (18)
Cl11—Pt1—K1—O13	−118.70 (4)	K1—O7—C8—C9	−30.9 (2)
Cl12^i^—Pt1—K1—O13	−47.88 (4)	O7—C8—C9—O10	67.2 (2)
Cl12—Pt1—K1—O13	132.12 (4)	C8—C9—O10—C11	−175.79 (19)
Cl11^i^—Pt1—K1—O1	168.03 (5)	C8—C9—O10—K1	−66.67 (19)
Cl11—Pt1—K1—O1	−11.97 (5)	O13—K1—O10—C9	−155.60 (15)
Cl12^i^—Pt1—K1—O1	58.86 (5)	O1—K1—O10—C9	116.38 (15)
Cl12—Pt1—K1—O1	−121.14 (5)	O7—K1—O10—C9	33.73 (13)
Cl11^i^—Pt1—K1—O7	−44.30 (4)	O16—K1—O10—C9	177.81 (13)
Cl11—Pt1—K1—O7	135.70 (4)	O4—K1—O10—C9	60.09 (14)
Cl12^i^—Pt1—K1—O7	−153.47 (4)	Cl11—K1—O10—C9	−95.81 (14)
Cl12—Pt1—K1—O7	26.53 (4)	Cl12—K1—O10—C9	−53.96 (13)
Cl11^i^—Pt1—K1—O16	115.33 (4)	Pt1—K1—O10—C9	−92.05 (14)
Cl11—Pt1—K1—O16	−64.67 (4)	O13—K1—O10—C11	−38.31 (12)
Cl12^i^—Pt1—K1—O16	6.16 (4)	O1—K1—O10—C11	−126.33 (14)
Cl12—Pt1—K1—O16	−173.84 (4)	O7—K1—O10—C11	151.03 (14)
Cl11^i^—Pt1—K1—O4	−114.81 (7)	O16—K1—O10—C11	−64.89 (13)
Cl11—Pt1—K1—O4	65.19 (7)	O4—K1—O10—C11	177.39 (12)
Cl12^i^—Pt1—K1—O4	136.02 (7)	Cl11—K1—O10—C11	21.48 (14)
Cl12—Pt1—K1—O4	−43.98 (7)	Cl12—K1—O10—C11	63.34 (12)
Cl11^i^—Pt1—K1—O10	4.97 (4)	Pt1—K1—O10—C11	25.24 (12)
Cl11—Pt1—K1—O10	−175.03 (4)	C9—O10—C11—C12	−179.93 (19)
Cl12^i^—Pt1—K1—O10	−104.20 (4)	K1—O10—C11—C12	67.74 (19)
Cl12—Pt1—K1—O10	75.80 (4)	O10—C11—C12—O13	−63.7 (3)
Cl11^i^—Pt1—K1—Cl11	180.0	C11—C12—O13—C14	−172.2 (2)
Cl12^i^—Pt1—K1—Cl11	70.83 (3)	C11—C12—O13—K1	20.3 (3)
Cl12—Pt1—K1—Cl11	−109.17 (3)	O1—K1—O13—C12	154.05 (15)
Cl11^i^—Pt1—K1—Cl12	−70.83 (3)	O7—K1—O13—C12	18.17 (17)
Cl11—Pt1—K1—Cl12	109.17 (3)	O16—K1—O13—C12	160.16 (17)
Cl12^i^—Pt1—K1—Cl12	180.0	O4—K1—O13—C12	84.84 (18)
O13—K1—O1—C18	9.94 (18)	O10—K1—O13—C12	9.66 (15)
O7—K1—O1—C18	145.56 (16)	Cl11—K1—O13—C12	−124.88 (16)
O16—K1—O1—C18	3.80 (16)	Cl12—K1—O13—C12	−64.12 (16)
O4—K1—O1—C18	150.31 (19)	Pt1—K1—O13—C12	−91.68 (16)
O10—K1—O1—C18	80.21 (19)	O1—K1—O13—C14	−12.20 (18)
Cl11—K1—O1—C18	−73.56 (17)	O7—K1—O13—C14	−148.08 (16)
Cl12—K1—O1—C18	−112.74 (17)	O16—K1—O13—C14	−6.10 (16)
Pt1—K1—O1—C18	−66.08 (18)	O4—K1—O13—C14	−81.41 (19)
O13—K1—O1—C2	−146.29 (18)	O10—K1—O13—C14	−156.59 (18)
O7—K1—O1—C2	−10.7 (2)	Cl11—K1—O13—C14	68.87 (17)
O16—K1—O1—C2	−152.4 (2)	Cl12—K1—O13—C14	129.63 (16)
O4—K1—O1—C2	−5.93 (17)	Pt1—K1—O13—C14	102.06 (17)
O10—K1—O1—C2	−76.0 (2)	C12—O13—C14—C15	169.0 (2)
Cl11—K1—O1—C2	130.20 (18)	K1—O13—C14—C15	−23.4 (3)
Cl12—K1—O1—C2	91.02 (19)	O13—C14—C15—O16	61.2 (3)
Pt1—K1—O1—C2	137.69 (17)	C14—C15—O16—C17	−177.82 (19)
C18—O1—C2—C3	177.7 (2)	C14—C15—O16—K1	−64.41 (19)
K1—O1—C2—C3	−24.0 (3)	O13—K1—O16—C17	152.14 (14)
O1—C2—C3—O4	62.9 (3)	O1—K1—O16—C17	−34.35 (12)
C2—C3—O4—C5	178.7 (2)	O7—K1—O16—C17	−121.28 (14)
C2—C3—O4—K1	−66.5 (2)	O4—K1—O16—C17	−63.50 (13)
O13—K1—O4—C5	−117.68 (14)	O10—K1—O16—C17	178.56 (12)
O1—K1—O4—C5	153.03 (15)	Cl11—K1—O16—C17	56.59 (12)
O7—K1—O4—C5	−32.10 (13)	Cl12—K1—O16—C17	87.15 (13)
O16—K1—O4—C5	−177.58 (13)	Pt1—K1—O16—C17	92.14 (12)
O10—K1—O4—C5	−58.19 (14)	O13—K1—O16—C15	35.36 (13)
Cl11—K1—O4—C5	98.30 (14)	O1—K1—O16—C15	−151.12 (15)
Cl12—K1—O4—C5	27.28 (14)	O7—K1—O16—C15	121.95 (14)
Pt1—K1—O4—C5	56.12 (16)	O4—K1—O16—C15	179.72 (13)
O13—K1—O4—C3	125.05 (14)	O10—K1—O16—C15	61.79 (14)
O1—K1—O4—C3	35.76 (13)	Cl11—K1—O16—C15	−60.19 (13)
O7—K1—O4—C3	−149.37 (15)	Cl12—K1—O16—C15	−29.62 (15)
O16—K1—O4—C3	65.15 (14)	Pt1—K1—O16—C15	−24.63 (13)
O10—K1—O4—C3	−175.46 (13)	C15—O16—C17—C18	177.84 (19)
Cl11—K1—O4—C3	−18.98 (15)	K1—O16—C17—C18	65.16 (19)
Cl12—K1—O4—C3	−89.99 (14)	C2—O1—C18—C17	−175.5 (2)
Pt1—K1—O4—C3	−61.15 (16)	K1—O1—C18—C17	26.0 (3)
C3—O4—C5—C6	178.7 (2)	O16—C17—C18—O1	−63.0 (3)

Symmetry codes: (i) −*x*+1, −*y*, −*z*+1.

### Hydrogen-bond geometry (Å, °)

**Table:**

*D*—H···*A*	*D*—H	H···*A*	*D*···*A*	*D*—H···*A*
C5—H5B···O10^ii^	0.99	2.57	3.318 (3)	133
C11—H11A···Cl11^i^	0.99	2.80	3.678 (3)	148
C17—H17A···Cl11	0.99	2.81	3.643 (3)	142

Symmetry codes: (ii) −*x*, *y*−1/2, −*z*+1/2; (i) −*x*+1, −*y*, −*z*+1.
